# Assessment of anxiety in parents of children undergoing tonsillectomy under general anaesthesia: A cross‐sectional study

**DOI:** 10.1002/hsr2.70087

**Published:** 2024-10-07

**Authors:** Khalid Maudood Siddiqui, Zohair Ahmed Khan, Muhammad Saad Yousuf, Muhammad Asghar Ali

**Affiliations:** ^1^ Department of Anaesthesiology Aga Khan University Karachi Pakistan; ^2^ Department of Anaesthesiology Aga Khan Health Services Gilgit Pakistan

**Keywords:** Amsterdam Preoperative Anxiety Information Scale (APAIS), parental anxiety, preoperative anxiety, tonsillectomy

## Abstract

**Background and Aims:**

Surgery in the pediatric age group entails a significant amount of anxiety for parents. Due to anxiety, parents are unable to take care of their children, which could affect the child's well‐being and contributes to poor outcomes. The primary objective of this study is to determine the frequency of preoperative anxiety in parents before the surgery of their children.

**Methods:**

It is a cross‐sectional descriptive study that included either parent of 147 children of American Society of Anaesthesiology (ASA) I & II, aged 1–12 years undergoing tonsillectomy over the period of 1 year. Each parent's demographic data were recorded and requested to answer a proforma containing the Amsterdam Preoperative Anxiety Information Scale (APAIS) for assessing anxiety on a 5‐point Likert scale. Median interquartile range (IQR), and frequency (%) were used to report the normal, skewed, and categorical variables. APAIS anxiety and information scores were compared by using either the Mann–Whitney *U*‐test or the Kruskal–Wallis test. Furthermore, anxiety scores were grouped (present/absent) with a cut‐off score of 11 for the presence of anxiety, and multivariate logistic regression was performed to explore the relationship between potential risk factors and the parent's anxiety.

**Results:**

Overall, anxiety was present in 59 (40.1%) respondents with 20 (33.9%) being fathers and 39 (66.1%) mothers. The median (IQR) for APAIS anxiety and information score were 9 ± 5 and 5 ± 2, respectively. Higher median anxiety scores were observed statistically significant in children under 5 years old, mother respondents, mothers aged 35 or younger, fathers under 40, and mothers with graduate or higher education (*p* < 0.05). Father respondents (AOR = 0.3, 95% CI = 0.1–0.8, *p* = 0.01), and mother's education less than graduation (AOR = 0.2, 95% CI = 0.1–0.6, *p* = 0.006) were also found to be statistically significant predictors.

**Conclusion:**

There is a significant prevalence of anxiety in parents of children who underwent surgery under general anaesthesia, and mothers have showed significantly higher anxiety levels than fathers.

## 1 INTRODUCTION

Surgery in the pediatric age group generally entails a significant level of anxiety for both the child and their parents. Parents are unable to effectively care for and support their children perioperatively because they become emotionally involved in the event. It is possible to determine the scope of the issue and devise procedures to enhance parent and child surgery experiences.^1^, ^2^ Physicians and nurses need to understand the impact of preoperative anxiety in parents on children and stated incidence of parents' anxiety extends from 50% to 75%.^3^, ^4^, ^5^

Approximately 47% of parents have been found to have an anxiety‐related illness. A variety of factors could play a role in perioperative parental anxiety, including parental separation, lack of control over the environment, and risk of complications or mortality. Parental gender, fear of postoperative pain, age of the child, and information about the anaesthesia were predictive factors associated with preoperative parental anxiety.^6^, ^7^

Despite the recognition of parental anxiety as an important determinant of the quality of recovery of the child postoperatively, there is a lack of regional data on the magnitude of its presence in our population. Therefore, it is essential to identify parental anxiety and devise interventions to allay it to improve the perioperative experience and outcome for the whole family.

The main objective of this study was to determine the frequency of preoperative anxiety in parents before the tonsillectomy of their children. Furthermore, the potential risk factors that caused parental anxiety were assessed.

## 2 MATERIALS AND METHODS

This cross‐sectional observational study was conducted in the surgical day care unit of Aga Khan University Hospital, Karachi, Pakistan. The institutional Ethics Review Committee (ERC) given an approval, with effect from April 16, 2019, over the period of 1 year (Ethical code: 2019‐1099‐3449).

### 2.1 Inclusion criteria

Either of child's parents with their children aged between 1 and 12 years with American Society of Anesthesiologists (ASA) I and II, undergoing tonsillectomy were included in the study. The ASA physical status classification system is a simple categorization of a patient's physiological status to help predict operative risks. It is a globally standardized classification system for preoperative condition documentation before surgery under anaesthesia.

### 2.2 Exclusion criteria

Parents with known psychological illness, those with a chronic debilitating disease like cognitive impairment, previous surgical exposure and parents who refused to enroll in the study were excluded. We also excluded the parents who had a language barrier (speaking only regional languages).

### 2.3 Ethical consideration

The study was approved by the ERC, and written Informed consent was obtained during preoperative assessment or at the preoperative holding area before surgery from parents. All children received standard care (received midazolam 0.5 mg/kg 30 min before surgery and accompanied to the operation theater along with one of their parents).

### 2.4 Study variables

**FIGURE 1:**
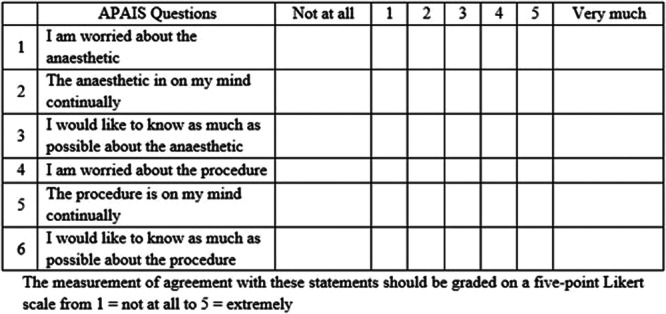
The Amsterdam Preoperative Anxiety and Information Scale (APAIS).

A standardized questionnaire pertinent to the study objective was used to collect information from the parents. The selection of parents was based on the availability and willingness to answer the questionnaire from an available parent. We have used an interpreter (proficient in English and Urdu language) to obtain the information. Supplementary information about demographics was taken from the patient's file. Preoperative anxiety was assessed by the investigator through the Amsterdam Preoperative Anxiety and Information Scale in the preoperative holding area (APAIS; Figure 1).^8^ The APAIS measures anxiety and the need‐for‐information with six items, with good reliability and validity.^9^ We also noted different variables which could have been associated with parent anxiety including (the child's age, child's gender, birth order of child, area of residence, parent's age, parent's gender, and parent's education). The questions were scored on a 5‐point Likert scale from 1 (not at all) to 5 (extremely). Sample size calculation was based on the study by Shirley et al.,^10^ who reported that large proportions of parents (42%) are anxious to the point of illness. Based on this study, 154 children undergoing tonsillectomy and their parents were included to estimate the target proportion with 95% confidence intervals within 8% margin of error.

All statistical analyses were conducted using R software (version 4.2.1, R Foundation for Statistical Computing, Vienna, Austria). First, the APAIS anxiety score (range = 4–20) was categorized into two groups (present/absent) with a cut‐off score of 11 for the presence of anxiety. Similarly, the APAIS information score (range = 2–10) was categorized into little/no information required (2–4), average information required, and high information required about the anesthetic and surgical procedure. Shapiro–Wilk test or Kolmogorov–Smirnov test was used to check the normality assumption of the quantitative variables such as the age of the child and parents and APAIS anxiety score etc. Normally distributed data were expressed as mean ± standard deviation and skewed data were reported as median with interquartile range (IQR). The categorical variables such as gender, respondents, residence, presence of anxiety, amount of information required, and birth order were reported in terms of frequency (%). To analyze the parent's anxiety and information scores, Kruskal–Wallis or Mann–Whitney (two‐sided) tests were applied. Multiple binary logistic regression model was performed to explore the relationship between potential risk factors and the primary outcome, that is, parent's anxiety. A p‐value less than 0.05 was a statistically significant level.

## 3 RESULTS

**FIGURE 2:**
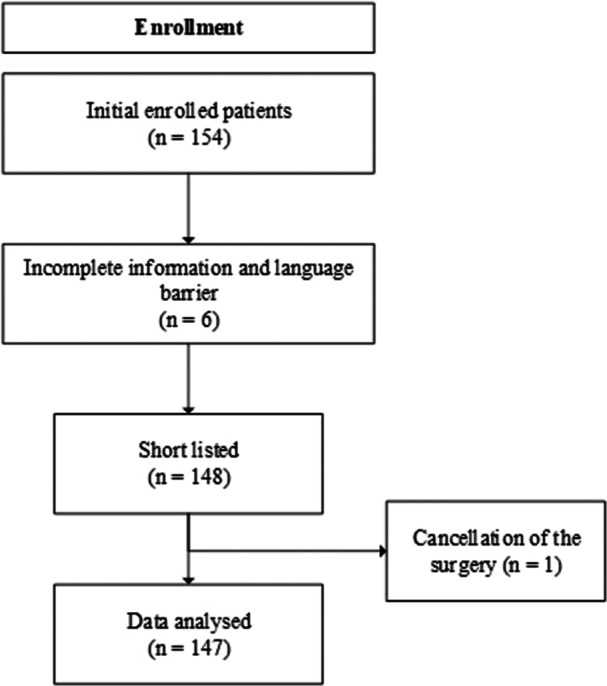
Flow chart of the study.

A total of 154 parents whose children were scheduled to undergo tonsillectomy were enrolled in the study. After excluding seven patients, data from 147 participants were available for the analysis (Figure 2). The median ± IQR for the age of the child, father, and mother were 7 ± 4, 38 ± 8, and 33 ± 8, respectively. Among the respondents, 75 (51%) were fathers and 72 (49%) were mothers. The median (IQR) or frequency (%) for the categorical demographic variables such as child's age, gender, birth order, respondent's gender, parent's age, education, and area of residence are shown in Table 1.

**TABLE 1:** Characteristics of the participants (*n* = 147).

Variables	Statistics
Age of the child (years)*	7 (4)
Age of the child for surgery (years)^a^	
<5	29 (19.7%)
5–10	94 (63.9%)
>10	24 (16.3%)
Gender^a^	
Female	63 (42.9%)
Male	84 (57.1%)
Respondent^a^	
Mother	72 (49.0%)
Father	75 (51.0%)
Birth Order of the child^a^	
First	78 (53.1%
Second	42 (28.6%)
Third and above	27 (18.4%)
Area of residence^a^	
Urban	132 (89.8%)
Rural	15 (10.2%)
Age of mothers*	33 (8)
Age of mothers^a^	
≤35	93 (63.3%)
>35	54 (36.7%)
Age of fathe**r** *	38 (8)
Age of father^a^	
<40	85 (57.8%)
≥40	62 (42.2%
Education of mother^a^	
Illiterate/primary or secondary	67 (45.6%)
Graduate and above	80 (54.4%)
Education of father^a^	
Illiterate/primary or secondary	38 (25.9%)
Graduate and above	109 (74.1%)

^a^ Results are presented as *n* (%).

* Median (IQR).

**FIGURE 3:**
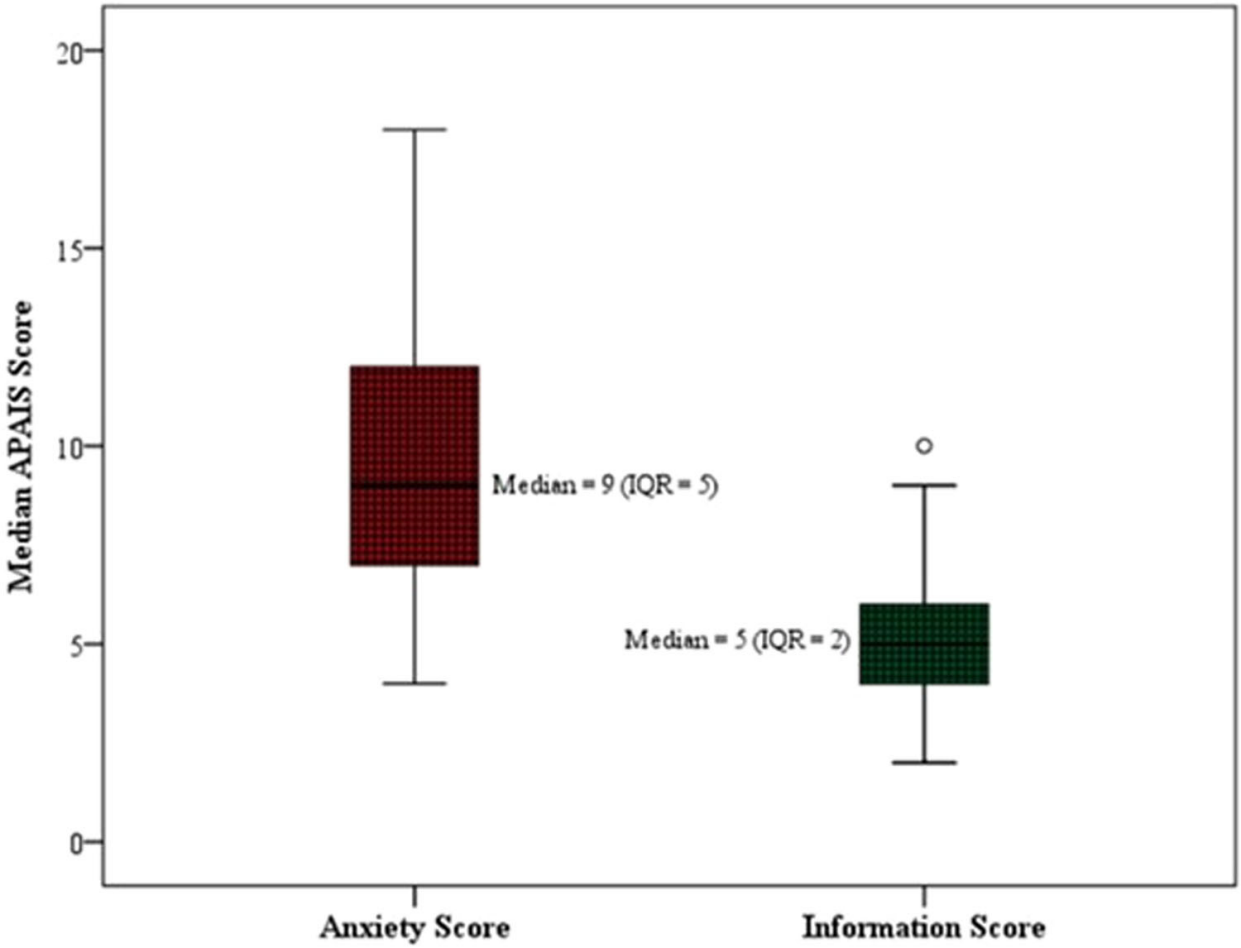
Median score of APIAS of parents before tonsillectomy in children (*n* = 147).

Overall, the median ± IQR APAIS anxiety score was 9 ± 5. The mothers were more anxious with an APAIS score of 11 ± 4.2 than the fathers 8 ± 5. (Figure 3). A statistically significant difference between parents' anxiety scores was observed among the factors such as the children under 5 years old (*p* = 0.004). Mothers experienced more anxiety than fathers (*p* < 0.001), particularly those aged 35 or younger (*p* = 0.01) and fathers under 40 (*p* = 0.02). Mothers with graduate or higher education showed increased anxiety (*p* = 0.002). Among information scores, only mothers' education was significantly associated, with higher scores in mothers with graduate or higher qualifications (*p* = 0.04) (Table 2). Similarly, for the information scores, Overall, the median ± IQR APAIS information score was 5 ± 2, and statistically significant differences between the scores were observed for the mother's education with a *p* = 0.04 (Table 2).

**TABLE 2:** Comparison of median anxiety and information score among factors (*n* = 147).

Variables	Parent's anxiety score		Information score	
	Median ± IQR	*p*‐value	Median ± IQR	*p*‐value
Age of the child for surgery (years)*				
<5	11.0 ± 4.0	**0.004**	4.0 ± 1.0	0.53
5–10	8.0 ± 5.0		5.0 ± 2.0	
>10	10.5 ± 5.2		5.0 ± 3.2	
Gender^a^				
Female	10.0 ± 5.0	0.21	5.0 ± 2.0	0.69
Male	9.0 ± 6.0		5.0 ± 2.2	
Respondent^a^				
Mother	11.0 ± 4.2	**<0.001**	5.0 ± 2.0	0.11
Father	8.0 ± 5.0		4.0 ± 2.5	
Birth order of the child*				
First	10.0 ± 5.0	0.12	5.0 ± 2.0	0.05
Second	10.0 ± 4.0		5.0 ± 2.8	
Third and above	8.0 ± 4.0		4.0 ± 1.5	
Area of residence^a^				
Urban	9.0 ± 5.2	0.90	5.0 ± 2.0	0.91
Rural	9.0 ± 3.0		5.0 ± 2.0	
Age of mothers^a^				
≤35	10.0 ± 6.0	**0.02**	5.0 ± 2.0	0.55
>35	8.0 ± 5.0		4.0 ± 2.0	
Age of father^a^				
<40	10.0 ± 6.0	**0.02**	5.0 ± 2.0	0.08
≥40	8.0 ± 5.0		4.0 ± 3.0	
Education of mother^a^				
Illiterate/primary or secondary	8.0 ± 5.0	**0.002**	4.0 ± 3.0	**0.04**
Graduate and above	11.0 ± 5.0		5.0 ± 3.0	
Education of father^a^				
Illiterate/primary or secondary	9.0 ± 6.2	0.69	4.0 ± 1.0	0.08
Graduate and above	9.0 ± 5.0		5.0 ± 2.0	

*Note*: A *p*‐values of less than 0.05 was considered as statistically significant.

* Kruskal–Wallis test for three categories.

^a^ Mann–Whitney for two categories.

After the categorization of the APAIS anxiety score, overall, the anxiety was present in 59 (40.1%) respondents with 20 (33.9%) being fathers and 39 (66.1%) mothers. Similarly, according to the APAIS information score, 67 (45.6%) respondents needed little information, 65 (44.2%) needed average, and 15 (10.2%) needed high information. The presence of anxiety was explored for any association with respondents' gender, child gender, child age group, parent's age group, and parents' educational level. After applying multivariable logistic regression, respondents' mother (AOR = 0.3, 95% CI = 0.1–0.8, *p* = 0.01), and the education of the mother (AOR = 0.2, 95% CI = 0.1–0.6, *p* = 0.006) were found to be statistically significant predictors (Table 3).

**TABLE 3:** Multivariate analysis showing the relationship between factors with parent's anxiety before tonsillectomy in children (*n* = 147).

Variables	Parent's anxiety *n* = 59	Multivariable analysis	
		AOR [95%CI]	*p*‐Value
Age of the child for surgery (years)			
<5	16 (27.1%)	0.7 (0.2–3.1)	0.67
5–10	31 (52.5%)	0.3 (0.1–1.1)	0.05
>10	12 (20.3%)	Ref.	
Gender			
Male	32 (54.2%)	0.9 (0.4–2.2)	0.87
Female	27 (45.8%)	Ref.	
Respondent			
Father	20 (33.9%)	0.3 (0.1–0.8)	0.01
Mother	39 (66.1%)	Ref.	
Birth order of the child			
First	35 (59.3%)	2.4 (0.7–0.4)	0.17
Second	19 (32.2%)	3.5 (0.9–14.0)	0.07
Third and above	5 (8.47%)	Ref.	
Area of residence			
Rural	5 (8.47%)	1.7 (0.4–7.0)	0.45
Urban	54 (91.5%)	Ref.	
Age of mothers			
≤35	42 (71.2%)	0.8 (0.3–2.4)	0.72
>35	17 (28.8%)	Ref.	
Age of father			
<40	41 (69.5%)	2.5 (0.9–7.5)	0.09
≥40	18 (30.5%)	Ref.	
Education of mother			
Illiterate/primary or secondary	17 (28.8%)	0.2 (0.1–0.6)	0.006
Graduate and above	42 (71.2%)	Ref.	
Education of father			
Illiterate/primary or secondary	15 (25.4%)	3.4 (1.1–12.8)	0.05
Graduate and above	44 (74.6%)	Ref.	

Abbreviation: AOR, adjusted odds ratio.

## 4 DISCUSSION

Tonsillectomy is one of the more common surgeries performed on children, and the procedure can cause substantial discomfort, bleeding, disruption of sleep, vomiting, and fever for a few days afterwards.

The primary aim of this study was to determine the prevalence of parental anxiety and the factors associated with it. Parental anxiety was measured using Amsterdam Preoperative Anxiety and Information Scale (APAIS), a questionnaire for assessing the preoperative anxiety of parents. The APAIS measures anxiety and the need‐for‐information with six items, with good reliability and validity.^9^ Overall, the percentage of parents who were anxious before the surgery of their child was 40.1%. A study by Ayenew and colleagues^5^ reported the prevalence of parental anxiety was 74.2%. In another previous study by Charana et al.,^11^ the parental anxiety levels were as high as 50%–74% amongst parents. The incidence of anxiety in our study is comparably low at 40%. The main reasons are probably lower literacy rate and strong family support in our country as compared to developed country. The age of the child, the parent's gender, information about the anaesthesia, and the education of the parents were the associated factors leading to parental anxiety. These associated factors are also consistent with our study.

Mothers were shown to be more anxious than fathers among parents, probably because mothers are more emotionally involved in raising their children and spend more time with their children and have higher trait anxiety and emotional sensitivity than fathers.^12^ Similar results have been reported by Norberg et al.^13^ and Scrimin et al.^14^

The age of the child has a significant impact on the parents' degree of anxiety, with parents of younger children experiencing higher levels of clinical anxiety. The difficulty in communicating with younger children may be a contributing factor to the higher anxiety experienced by parents of younger children. This finding was consistent with other studies. Additionally, Rosenberg et al.^15^ found that parents' psychological factors such as poor coping, low self‐efficacy, and external loss of control associated with higher parent preoperative anxiety in young children. The study also identified that higher parental preoperative anxiety was linked to higher infant postoperative pain scores in the first 24 h.

In contrast to this, previous research revealed that older children typically feel fear, rage, and guilt before surgery in the waiting room; these issues with the child likely to develop clinical anxiety among parents. Older children are more anxious about the procedure than younger children, who will keep quiet; thus, the existence of separation anxiety in both the child and the parent may cause the parent's anxiety. As a result, child anxiety may cause parent anxiety, and vice versa.^16^ The current study has found that parents' literacy levels and geographic locations are predictors of their anxiety of parents regarding anaesthesia and surgery. Urban parents with higher levels of education reported feeling more anxious.^17^ These findings were also observed in a study by Litman and colleagues,^18^ who observed that parents experience greater apprehension when their child is under a year old and when it is the child's first surgery.

Education level is one of a frequently mentioned predictor of preoperative parental anxiety. Güzel et al.^19^ demonstrated that a higher level of education has a link with higher anxiety scores. It has been demonstrated that highly educated individuals tend to be more extroverted, engage in information‐seeking activity, and are aware of the possible hazards associated with surgery. Parents with lower levels of education are thought to experience less anxiety, which can be explained by the fact that less educated people had a limited understanding of anaesthesia and surgery. Our study was conducted in a private tertiary care center of a metropolis and most of the participants were well‐educated and belonged to the city. According to the multivariate analysis, only parent's gender and mother's education were associated with parental anxiety. Other factors, such as the age of the child and urban/rural residence, were not such associations.

Both the type of surgery and the parent's level of education had little impact on how well the information was perceived. This is crucial because it demonstrates that although scars, complications, and length of stay may seem to be the most crucial aspects of preoperative information, parents also need to be aware of practical information like visiting hours and how to manage chronic medications. Having a hospital information booklet or having a nurse go over all these details greatly enhances the experience of parents. Finally, having the parents' input on what information they need, rather than only relying on our practice on our medical societies' recommendations of what information should be given, is essential and contributes to the global shift towards patient‐centered outcomes.^20^

One interesting thing we have found is that our study population is slightly skewed towards the urban, educated middle‐class families who can afford private care; it may not account for a significantly lower frequency among families not captured adequately in our observations. Therefore, it is important to conduct multi‐center studies to determine the prevalence of parental anxiety more precisely in the perioperative period, while at the same time developing strategies to allay this phenomenon so that the perioperative experience is less traumatic and does not lead to iatrogenic suffering for the whole family afterwards.

The strength of this study is the prevalence of anxiety and it's contributing causes which are never investigated before in a low middle‐income country. Parents investigated in this study were those whose children were undergoing one type of surgery. As a result, it was possible to investigate how a particular diagnosis affects the severity of anxiety. Tonsillectomy as a surgical procedure has been chosen not only as a common daycare procedure but also to maintain uniformity in surgical procedures and reduce the confounding factors that could be added if we selected random procedures; this is also one of its strengths. Another strength of the study, the use of APAIS scale, which is highly correlated to the STAI‐State questionnaire, supporting the validity of APAIS in measuring anxiety status. It can be completed in less than 2 min, and its stability in patients. Responses which were highlighted with test–retest reliability make it a very useful tool in the assessment of preoperative anxiety in clinical practice. Since APAIS is quicker and easier to administer than STAI, it might be a good alternative for measuring preoperative anxiety.^21^

In addition, the degree of parental anxiety may vary depending on a particular time and place throughout the preoperative period. This study is conducted in a single private tertiary care center and findings cannot be extrapolated to other private and public sector hospitals. We didn't measure children's anxiety, which is one of the influential factors in parents' anxiety. That could be considered as the weakness of the study. Another limitation is that other parents (mother or father) who were not included in this study might be more anxious, which could also influence the study results. Furthermore, for administering the APAIS questionnaire, the interpreters were used to guide parents; therefore, it could affect the results of the study. These factors are some of the limitations of this study. The implementation of interventions to reduce parental anxiety over their child's operation is highly supported for the benefit of the child's health, the parent's health, and the healthcare system. Parents frequently accompany children in the waiting area, preoperative holding room, and post‐anaesthesia care unit since they are important partners for healthcare professionals during the perioperative period. Parents' satisfaction with care, the success of children's surgeries, and the effectiveness of care on the day of surgery could all be significantly improved by educating and preparing parents to effectively manage their anxieties.

## 5 CONCLUSION

It was observed that there was a significant prevalence of anxiety in parents of children who underwent tonsillectomy with disparity in anxiety levels between the child's mother and father, with mothers showing significantly higher anxiety levels than fathers. It is suggested to develop preoperative programs and counseling sessions for families to mitigate their fears and improve outcomes. The anaesthesiologist's preoperative consultation can help in clarifying the facts related to the operation and calming parents.

## AUTHOR CONTRIBUTIONS

**Khalid Maudood Siddiqui**: Conceptualization; methodology; supervision; writing—original draft. **Zohair Ahmed Khan**: Investigation; software; data curation. **Muhammad Saad Yousuf**: Validation; data curation; investigation. **Muhammad Asghar Ali**: Visualization; writing—review and editing.

## CONFLICT OF INTEREST STATEMENT

The authors declare no conflict of interest. There was no involvement of a supporting source or financial relation.

## TRANSPARENCY STATEMENT

The lead author Khalid Maudood Siddiqui affirms that this manuscript is an honest, accurate, and transparent account of the study being reported; that no important aspects of the study have been omitted; and that any discrepancies from the study as planned (and, if relevant, registered) have been explained.

## Data Availability

The data that support the findings of this study are available from the corresponding author upon reasonable request.
